# Application of Microwave-Assisted Water Extraction (MAWE) to Fully Realize Various Physiological Activities of *Melaleuca quinquenervia* Leaf Extract

**DOI:** 10.3390/plants13233362

**Published:** 2024-11-29

**Authors:** Ting-Kang Lin, Jyh-Yih Leu, Yi-Lin Lai, Yu-Chi Chang, Ying-Chien Chung, Hsia-Wei Liu

**Affiliations:** 1 Graduate Institute of Applied Science and Engineering, Fu Jen Catholic University, New Taipei City 242062, Taiwan; dingganglin@gmail.com (T.-K.L.); yihleu@gmail.com (J.-Y.L.); 2 Department of Life Science, Fu Jen Catholic University, New Taipei City 242062, Taiwan; 3 Department of Biological Science and Technology, China University of Science and Technology, Taipei City 115311, Taiwan; yilinlai519@gmail.com (Y.-L.L.); yuchi0618@cc.cust.edu.tw (Y.-C.C.)

**Keywords:** antioxidant, cytotoxicity, microwave-assisted extraction, *Melaleuca quinquenervia*, molecular docking

## Abstract

*Melaleuca quinquenervia* is widely grown in tropical areas worldwide. Studies have demonstrated that extracts of its buds, leaves, and branches obtained through hydrodistillation, steam distillation, or solvent extraction exhibit physiological activities, including anti-melanogenic, antibacterial, and antioxidant properties; nevertheless, such extracts are mostly not effectively collected or adequately utilized. Accordingly, this study applied a rapid, effective, and easy-to-operate microwave-assisted water extraction (MAWE) technique for the first time to prepare *M. quinquenervia* leaf extract (MLE) with improved physiological activities. The results indicated that the optimal irradiation time and liquid/solid ratio for the production of the MLE were 180 s and 20 mL/g, respectively. Under optimal conditions, the freeze-dried MLE achieved a high yield (6.28% ± 0.08%) and highly effective broad-spectrum physiological activities. The MLE exhibited strong antioxidant, antiaging, and anti-inflammatory activities and excellent antityrosinase and antimicrobial activities. Additionally, the MLE was noncytotoxic at concentrations of ≤300 mg/L, at which it exhibited pharmacological activity. The results also indicated that the MLE comprised a total of 24 chemical compounds and 17 phenolic compounds. Among these compounds, luteolin contributed to antityrosinase activity. The extract’s antiaging activity was attributed to ellagic acid and quercetin, its anti-inflammatory activity resulted from ellagic acid and kaempferol, and its antimicrobial activity resulted from quercetin and 3-*O*-methylellagic acid. In conclusion, the MAWE-derived MLE may be useful as a functional ingredient in cosmetic products, health foods, and botanical drugs.

## 1. Introduction

*Melaleuca quinquenervia* is extensively grown in Taiwan and is often used as a street tree, landscape tree, and windbreak [1]. *M. quinquenervia* belongs to a class of large evergreen trees characterized by protruding nodules on the trunk and brown or off-white bark. The leaves and buds of *M. quinquenervia* contain essential oils, which are often extracted for use as antibacterial agents, preservatives, analgesics, pesticides, tranquilizers, and treatments for atopic dermatitis or eczema [2,3,4]. Therefore, *M. quinquenervia* leaf and bud extracts are widely used as the main raw material in daily chemicals, beauty products, and health-care products. Although *M. quinquenervia* leaf extract (MLE) exhibits physiological activities, including skin-whitening, antibacterial, antiaging, and anti-inflammatory effects, this extract is mostly not effectively collected or adequately utilized [1]. This is primarily attributed to the lack of effective and appropriate extraction techniques. Hence, developing appropriate extraction techniques can improve the commercial value and pharmacological activity of MLEs.

Common techniques for extracting essential oils from plants include hydrodistillation, steam distillation, cold pressing, enfleurage, and solvent extraction [5]. Hydrodistillation and steam distillation are easy to operate, but these techniques provide low yields of essential oils [6]. Cold pressing is suitable only for extracting essential oils from citrus peels [7]. Enfleurage is a traditional extraction technique with complicated and labor-intensive procedures [8]; hence, it is mainly used to extract essential oils from flowers for producing perfumes and balms. Solvent extraction entails placing plants in organic solvents (e.g., ether, hexane, toluene, formaldehyde, acetaldehyde, or alcohol) in a closed container for extraction; this technique achieves a higher yield of essential oils than does distillation, but it requires a longer extraction time and can extract only chemical compounds of specific polarity [5]. In recent years, scholars have developed supercritical fluid extraction, ultrasound-assisted extraction, and microwave-assisted extraction (MAE) techniques to improve the extraction efficiency and shorten the extraction time [9]. Among these techniques, MAE is particularly noteworthy for its effectiveness in minimizing extraction time while simultaneously improving the yield of phenolic compounds [10]. Additionally, MAE is recognized as an energy-saving, environmentally friendly, and easy-to-operate technique [10]. Compared with hydrodistillation, MAE yields extracts with lower degrees of thermal decomposition and oxidation; thus, in MAE-derived extracts, the active ingredients in natural products are preserved. Furthermore, relative to hydrodistillation, MAE can shorten the extraction time by 5–20 times and can produce a greater yield of active compounds through the rapid breakdown of cell walls and extraction of intracellular substances [10].

Steam distillation and solvent (80% ethanol, water, and methanol) extraction techniques have been used to derive extracts from different parts of *M. quinquenervia* trees (e.g., buds, leaves, and branches) [1,2,11]. Such extracts have been reported to demonstrate various physiological activities, including antioxidant, anti-melanogenic, antigenotoxic, hypoglycemic, antifungal, and anti-inflammatory effects, in addition to exhibiting insecticidal activity against mosquitoes [1,2,11]. However, the types and concentrations of active ingredients in the extracts can vary according to the region of harvest, type of extraction technique used, or part of the plant used for extraction. Accordingly, the aim of the present study was to determine the physiological activities of an MLE derived through microwave-assisted water extraction (MAWE) in order to maximize the commercial value of the extract.

## 2. Results and Discussion

### 2.1. Optimization of MAE Conditions

**Figure 1:**
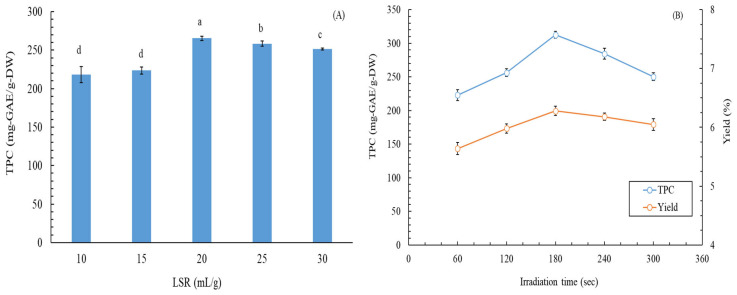
(**A**) Effects of LSR on the TPC of the MLE when the irradiation time was 120 s and extraction temperature was 80 °C. (**B**) Effects of irradiation time on the TPC and yield of the MLE when the LSR was 20 and the extraction temperature was 80 °C. Data are expressed as means and standard deviations of three independent experiments. The lowercase letters in the subfigure A indicate significant differences at the *p* < 0.05 level.

Previous studies have reported a positive correlation between total phenolic content (TPC) and antioxidant activity [12]. Therefore, in this study, the TPC of the *M. quinquenervia* leaf extract (MLE) was used as an indicator to optimize MAWE conditions. Figure 1A illustrates the effect of various liquid/solid ratios (LSRs) on the TPC of the MLE. As the LSR changed, the TPC of the MLE first increased and then decreased, and the highest TPC (265.4 ± 2.8 mg gallic acid equivalent (GAE)/g dry weight (DW)) was observed at an LSR of 20 mL/g. Figure 1B presents the effects of irradiation time on the TPC and yield of the MLE. The TPC and yield of the MLE varied with irradiation time. The optimal TPC and yield of the MLE (312.7 ± 5.2 mg GAE/g DW and 6.28 ± 0.08%, respectively) were observed at an irradiation time of 180 s. The TPC of the MLE in this study was determined to be superior to those of methanol leaf extract of *M. cajuputi* (37 ± 0.02 mg GAE/g DW) [13], ethanol extract of *M. bracteata* (88.6 ± 1.3 mg GAE/g DW) [14], ethanol flower extract of *M. leucadendron* (153.8 ± 1.9 mg GAE/g DW) [15], and butanol extract of *M. leucadendron* (289.23 ± 5.21 mg GAE/g DW) [16]. In addition, the yield of the MLE in this study was determined to be considerably superior to that of butanol extract of *M. leucadendron* (2.22%) [16]; it was also superior to those of hydrodistillation extract of *M. leucadendra* (0.75%) [17], hydrodistillation extract of *M. leucadendra* (0.7%) [18], and hydrodistillation extract of young (1.22%) and old (1.43%) *M. leucadendra* leaves [19]. Although experimental data for the same species were not available for comparison with our results, we clearly determined the application potential of the MAWE technique for obtaining *M. quinquenervia* extracts with high antioxidant activity and yield.

### 2.2. Extracellular and Intracellular Antityrosinase Activity

**Figure 2:**
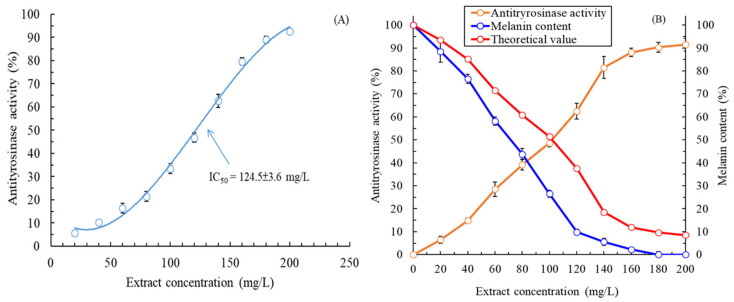
(**A**) Extracellular antityrosinase activity and (**B**) intracellular antityrosinase activity and melanin content in HEMn cells after treatment with the MLE obtained through MAWE (operating conditions: LSR, 20 mL/g; extraction temperature, 80 °C; microwave irradiation time, 180 s; and microwave irradiation power, 700 W). Data are expressed as means and standard deviations of three independent experiments.

The antityrosinase activity of the MLE was evaluated under optimal extraction conditions (LSR: 20 mL/g; irradiation time: 180 s). The extracellular and intracellular antityrosinase activities of the MLE are displayed in Figure 2A,B, respectively. The antityrosinase activity of the MLE gradually increased with the concentration of the extract. The half-maximal inhibitory concentration (IC_50_) value derived for the intracellular antityrosinase activity of the extract was 124.5 ± 3.6 mg/L, which was noted to be superior to those of α-arbutin (161.8 ± 5.4 mg/L), aqueous methanolic leaf extract of *Melaleuca subulata* (287.04 ± 4.19 mg/L) [20], and polyphenol-rich fraction of *Melaleuca rugulosa* leaves (200.56 ± 2.08 mg/L) [21]. However, the IC_50_ value derived for the antityrosinase activity of the MLE was noted to be inferior to that of kojic acid (positive control; 14.5 ± 0.8 mg/L). Figure 2B reveals a nonproportional relationship between a decrease in melanin content in the HEMn cells and an increase in the intracellular antityrosinase activity of the MLE. The results indicated that the mechanism underlying the whitening effects of the MLE (in terms of the melanin content in the HEMn cells) was complex and did not involve only the inhibition of tyrosinase activity. The mechanism might also involve the inhibition and regulation of other melanogenic genes or enzymes [22]. In addition, the IC_50_ value derived for the intracellular antityrosinase activity of the extract was 102.1 ± 1.8 mg/L, which was superior to the value found for intracellular antityrosinase activity. In this study, the MAWE-derived MLE exhibited significantly effective antityrosinase activity; this is because the melanin content decreased to 9.8 ± 1.2% in the HEMn cells treated with the MLE at a low concentration (120 mg/L).

### 2.3. Cytotoxicity Assay

**Figure 3:**
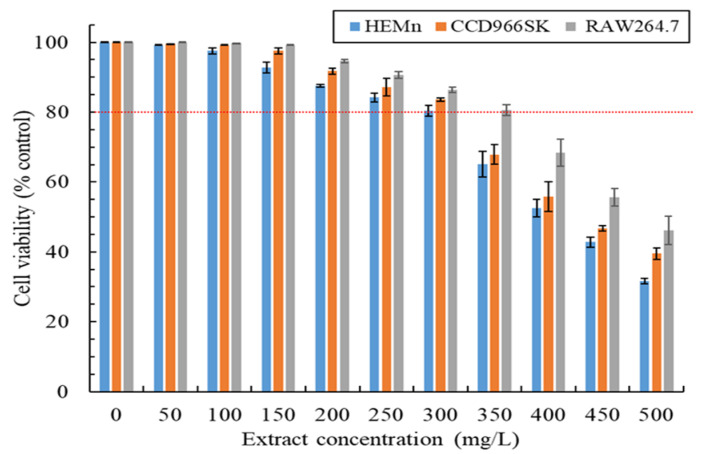
Cytotoxic effects of the MLE obtained through MAWE on HEMn, CCD966SK, and RAW264.7 cells after exposure for 24 h (operating conditions: LSR, 20 mL/g; extraction temperature, 80 °C; microwave irradiation time, 180 s; and microwave irradiation power, 700 W). Data are expressed as means and standard deviations of three independent experiments. The red dashed line indicates 80% cell viability.

In product development, product functionality is crucial, but product safety also warrants consideration. Accordingly, this study assessed the viability of the HEMn, CCD966SK, and RAW264.7 cells treated with the MLE at different concentrations, and the results are illustrated in Figure 3. Cell viability decreased as the MLE concentration increased. Among the tested cells, the HEMn cells were the most sensitive to the cytotoxic effects of the MLE, followed by the CCD966SK and RAW264.7 cells. Higher cell viability indicates that the extract is safer. Generally, cell viability of >80% implies that the extract is nontoxic [23]. In this study, the safe doses or concentrations of the MLE for the HEMn, CCD966SK, and RAW264.7 cells were found to be 300, 300, and 350 mg/L, respectively. The IC_50_ values derived for the cytotoxic effects of the MLE against the HEMn, CCD966SK, and RAW264.7 cells were 392.4 ± 5.1, 431.8 ± 7.6, and 482.3 ± 2.5 mg/L, respectively. According to these findings, the safety of the MAWE-derived MLE can be considered to be much superior to that of *M. quinquenervia* extract obtained through steam distillation [1], *M. 1eucadendron* extract obtained through hydrodistillation [17], ethanol leaf extract of *M. 1eucadendron* [24], and butanol extract of *M. Leucadendron* [16]. The low cytotoxicity of the MLE may be because the MAWE technique uses water as the extraction solvent, indicating the promising safety of MAWE for plant extraction.

### 2.4. Evaluation of Antioxidant Activity

**Figure 4:**
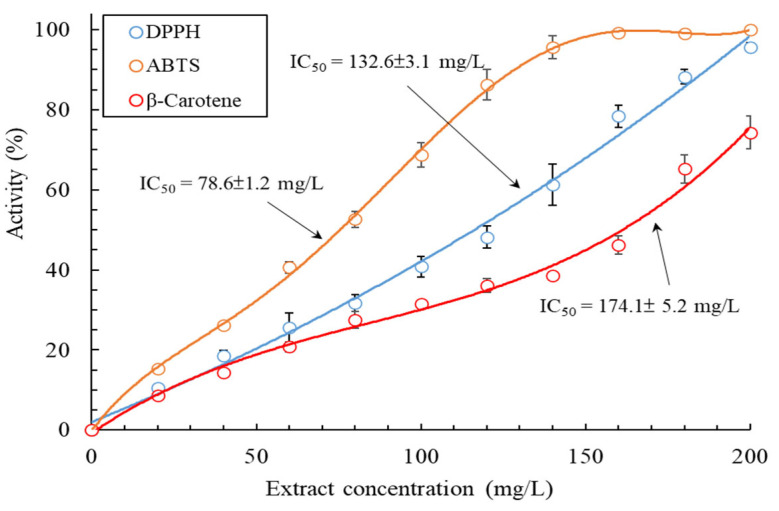
Antioxidant activity of the MLE obtained through MAWE (operating conditions: LSR, 20 mL/g; extraction temperature, 80 °C; microwave irradiation time, 180 s; and microwave irradiation power, 700 W). Data are expressed as means and standard deviations of three independent experiments.

A single indicator cannot be used for examining antioxidant activity/capacity, particularly for multifunctional or complex multiphase systems, because several variables affect antioxidant activity [25]. In addition, the MLE may contain hydrophilic and hydrophobic compounds. Hence, in this study, the TPC, total flavonoid content (TFC), 2,2-diphenyl-1-picrylhydrazyl (DPPH) radical scavenging activity, 2,2′-azino-bis(3-ethylbenzothiazoline-6-sulfonic acid) (ABTS) radical scavenging activity, and *β*-carotene bleaching (BCB) activity of the MLE were evaluated to provide comprehensive insights into the mechanisms underlying the antioxidant activity of the extract. The TPC and TFC of the MLE were 312.7 ± 5.2 mg GAE/g DW and 71.3 ± 0.2 mg rutin equivalents (RE)/g DW, respectively. As mentioned, the TPC of the MLE derived through the MAWE technique was superior to that derived through solvent extraction. Similarly, the TFC of the MLE was much superior to that of ethanol extract of *M. bracteate* (19.4 mg RE/g DW) [14]. Figure 4 presents the DPPH radical scavenging activity, ABTS radical scavenging activity, and BCB activity of the MLE. These antioxidant activities of the MLE increased with the concentration of the extract. The IC_50_ values derived for DPPH scavenging, ABTS scavenging, and BCB activities were 132.6 ± 3.1, 78.6 ± 1.2, and 174.1 ± 5.2 mg/L, respectively, which were inferior to the values derived for the positive control (ascorbic acid: 18.6 mg/L for DPPH scavenging activity) and butylated hydroxytoluene (BHT) (82.6 mg/L for ABTS scavenging activity and 12.4 mg/L for BCB activity).

The IC_50_ values derived for DPPH scavenging activity for the MLE were noted to be inferior to those observed for a polyphenol-rich fraction of *M. rugulosa* leaves (22.05 mg/L) [21], aqueous methanolic leaf extract of *M. subulata* (12.0 mg/L) [20], ethanol leaf extract of *M. 1eucadendron* (7.32 mg/L) [24], and butanol extract of *M. 1eucadendron* (5.1 mg/L) [16]. However, the IC_50_ values derived for the MLE were significantly superior to those derived for hydrodistillation-based leaf extract of *M. 1eucadendron* (2400 mg/L) [18]. These results indicate that polar solvent extraction is beneficial for extracting substances with antioxidant activity from *Melaleuca* leaves. In addition, the IC_50_ values derived for BCB activity for the MLE were determined to be inferior to those derived for a polyphenol-rich fraction of *M. rugulosa* leaves (11.31 mg/L) [21] and aqueous methanolic leaf extract of *M. subulata* (4.31 mg/L) [20]. These findings demonstrate that the MLE derived through MAWE exhibited hydrophilic and hydrophobic antioxidant activities, indicating that the MAWE technique can be used in various applications. The chemical composition and relative content of the MLE are provided in the subsequent sections.

### 2.5. Evaluation of Antiaging Activity

Antioxidant activity/capacity is a comprehensive indicator of the physiological activities of plant extracts [25]. To assess the antiaging activity of extracts, the activity of aging-related enzymes must be evaluated. Matrix metalloproteinase-1 (MMP-1), collagenase, elastase, and hyaluronidase catalyze the degradation of the main components of the extracellular matrix. Thus, their activities in the skin indicate the current condition of the skin (wrinkles, elasticity, and even luster). Table 1 presents the IC_50_ values derived for the antiaging activities of the MLE. The IC_50_ values derived for the inhibitory effects of the MLE on MMP-1, collagenase, elastase, and hyaluronidase activities were 114.8 ± 8.1, 187.2 ± 5.4, 73.4 ± 2.4, and 60.4 ± 3.1 mg/L, respectively. The IC_50_ value derived for the inhibitory effect of the MLE on MMP-1 activity was inferior to that of EGCG (positive control; 42.3 ± 3.1 mg/L). Moreover, the IC_50_ value derived for the inhibitory effect of the MLE on collagenase activity was slightly inferior to those of EGCG (positive control; 113.7 ± 9.1 mg/L) and gallic acid (positive control; 126.8 ± 3.7 mg/L). The IC_50_ value derived for the inhibitory effect of the MLE on elastase activity was relatively close to those of EGCG (93.5 ± 7.2 mg/L) and oleanolic acid (positive control; 78.2 ± 1.8 mg/L). However, the IC_50_ value derived for the inhibitory effect of the MLE on hyaluronidase activity was significantly superior to those of the commercial antiaging agent epigallocatechin gallate (EGCG) (382 ± 12.6 mg/L) and oleanolic acid (98.6 ± 5.2 mg/L). The results may be attributed to the various active phytoconstituents of the MLE.

We compared our results with those of previous studies and observed that the IC_50_ values derived for the inhibitory effect of the MLE on collagenase activity were significantly superior to those of a polyphenol-rich fraction of *M. rugulosa* leaves (410 mg/L) [21] and aqueous methanolic leaf extract of *M. subulata* (382.16 mg/L) [20]. However, the IC_50_ values derived for the inhibitory effect of the MLE on elastase activity were slightly inferior to those of a polyphenol-rich fraction of *M. rugulosa* leaves (68.18 mg/L) [21] and aqueous methanolic leaf extract of *M. subulata* (59.18 mg/L) [20]. Although limited data on the antiaging activity of *M. quinquenervia* extracts were available for comparison, the data for the extracts from the same genus of *Melaleuca* indicate that the MLE obtained through MAWE has considerable potential for commercial application owing to its antiaging activity.

### 2.6. Evaluation of Anti-Inflammatory Activity

**Figure 5:**
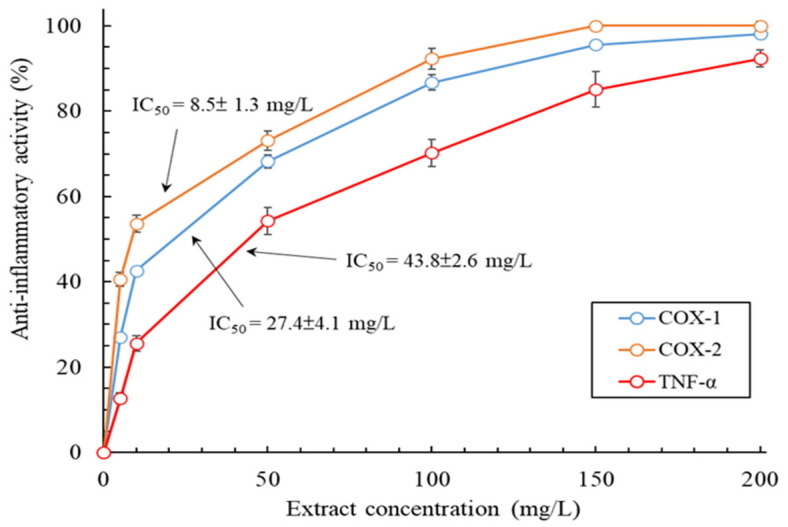
Anti-inflammatory activity of the MLE obtained through MAWE (operating conditions: LSR, 20 mL/g; extraction temperature, 80 °C; microwave irradiation time, 180 s; and microwave irradiation power, 700 W). Data are expressed as means and standard deviations of three independent experiments.

Extracts with anti-inflammatory activity could be a potential ingredient in skin care products, health products, and even pharmaceutical products. Accordingly, this study examined the anti-inflammatory activity of the MLE, and the results are displayed in Figure 5. Specifically, the inhibitory effects of the MLE on cyclooxygenase-1 (COX-1) activity, cyclooxygenase-2 (COX-2) activity, and tumor necrosis factor-α (TNF-α) production increased exponentially as the extract’s concentration was increased. The inhibitory effects of the MLE at a concentration of 200 mg/L on COX-1 activity, COX-2 activity, and TNF-α production were 98.1 ± 0.8%, 100 ± 0.2%, and 92.3 ± 2%; this concentration was not cytotoxic to skin cells. In addition, the IC_50_ values derived for the inhibitory effects of the MLE on COX-1 activity, COX-2 activity, and TNF-α production were 27.4 ± 4.1, 8.5 ± 1.3, and 43.8 ± 2.6 mg/L, respectively. The inhibitory effects of the MLE were strongest for COX-2 activity. The IC_50_ value derived for the inhibitory effects of the anti-inflammatory drug indomethacin (positive control) on COX-1 activity, COX-2 activity, and TNF-α production were 5.2 ± 0.4, 43.6 ± 3.7, and 6.52 ± 0.8 mg/L, respectively. The anti-inflammatory activity of the MLE was slightly inferior to that of the highly purified polyphenol-rich fraction of *M. rugulosa* leaves [21]. Nevertheless, the anti-inflammatory activity of the MLE meets the requirements for commercial medicinal use.

### 2.7. Evaluation of Antimicrobial Activity

In accordance with regulatory guidelines for USP 51 antimicrobial effectiveness testing and considering the microbes associated with skin diseases, this study evaluated the antimicrobial activity of the MLE. Table 2 lists the minimum inhibitory concentration (MIC) values derived for the activity of the MLE against *S. aureus*, *E. coli*, *P. aeruginosa*, and *C. acnes* strains and the minimum fungicidal concentration (MFC) values derived for the activity of the MLE against *C. albicans* and *A. brasiliensis* strains. The MIC and MFC values derived for the activity of the MLE against the tested bacterial and fungal strains were 64–128 and 128–256 mg/L, respectively. The concentrations at which the MLE exhibited antimicrobial activity were not cytotoxic to skin cells (Figure 3). The MLE exhibited stronger antibacterial activity against *S. aureus* than it did against *C. acnes.* This finding is consistent with that of Shakeel et al. (2021), who used essential oils extracted from *M. quinquenervia* [3]. The MLE exhibited strong antifungal activity against the *C. albicans* and *A. brasiliensis* strains. Valková et al. (2022) reported that essential oils extracted from *M. quinquenervia* leaves through steam distillation were effective against various *Penicillium* strains [4]. Notably, the antimicrobial activity of the MLE was significantly stronger than that of leaf extracts of similar species (*M. leucadendra*) [26,27,28]. These results indicate that the MLE obtained through MAWE has excellent and broad-spectrum antimicrobial activity. A possible reason for the excellent antimicrobial activity of the MLE is the use of microwaves in MAWE to improve the effectiveness and efficiency of cell breakdown [9], which could result in the release of various antimicrobial ingredients. These findings demonstrate that the MLE has favorable cosmeceutical properties. In addition to its high antimicrobial activity, the MLE has the potential to become a valuable ingredient in skin care products; its use in such products can ameliorate the necessity for the addition of artificial preservatives. In addition, it can be applied as an antimicrobial agent in cleaning products, the food industry, and even in medicine.

### 2.8. Chemical Composition of MLE

This study subjected the MLE to gas chromatography–mass spectrometry (GC-MS) and high-performance liquid chromatography (HPLC) to determine its primary constituents and major phenolic compounds, respectively. Table 3 lists the primary constituents and their relative content associated with the volatile part of the MLE. This table lists only constituents whose relative content in the volatile part of the MLE was >0.5%; these constituents constituted 98.44% of the volatile part of the MLE. The volatile part of the MLE had a total of 24 components, and compounds with a relative content of >7% were as follows: 1,8-cineole (16.71%), viridiflorol (13.27%), α-pinene (10.61%), α-terpineol (10.12%), ledene (8.52%), limonene (7.94%), and β-pinene (7.52%). The main components in the MLE, especially 1,8-cineole, viridiflorol, and α-terpineol, were noted to be similar to those identified by Chao et al. (2017) [1], Valková et al. (2022) [4], and Vázquez et al. (2023) [29], but the relative content of these components was different from those identified by them. In this study, monoterpene was the dominant component (63.18%), followed by sesquiterpene (32.07%), aromatic compounds (2.45%), and hydrocarbons (0.74%). Studies have also reported that monoterpene compounds were the most abundant (25.42–62.4%) components of MLEs, regardless of the applied extraction technique [1,4,29].

The main phenolic compounds in plants are phenolic acids and flavonoids, which include flavonols, anthocyanins, and isoflavones [30]. Table 4 lists the main phenolic compounds and their relative content in the MLE. A total of 17 phenolic compounds were identified in the MLE: 7 phenolic acids and 10 flavonoids. The predominant compounds in the MLE were gallic acid, ellagic acid, 3-*O*-methylellagic acid, luteolin, kaempferol, and quercetin. The total concentrations of phenolic acids (304.1 mg GAE/g DW) and flavonoids (70.1 mg RE/g DW) were close to those of the TPC (312.7 mg GAE/g DW) and TFC (71.3 mg RE/g DW) of the MLE. These findings demonstrate that most phenolic compounds in the MLE were detected in this study. Gallic acid, ellagic acid, and 3-*O*-methylellagic acid were also detected in ethanol leaf extract of *M. quinquenervia* [2]. The possible roles of these components in the pharmacological activities of the MLE are discussed in the subsequent section.

### 2.9. Molecular Docking Analysis

In this investigation, a total of 41 distinct compositions within MLE were identified. Previous studies indicated that extracts with a higher concentration of active components exhibited a more pronounced inhibitory effect on enzyme activities. Consequently, this study conducted a molecular docking analysis of the 10 main phytocompounds in the MLE against representative enzymes or proteins. The evaluation of whitening activity was conducted through the assessment of binding affinity between the compounds and tyrosinase. Similarly, antiaging activity was gauged by the binding affinities of the compounds with elastase, collagenase, hyaluronidase, and MMP-1. Anti-inflammatory activity was assessed based on the binding affinities with COX-1, COX-2, and TNF-α, while antimicrobial activity was evaluated through the binding affinities with tyrosyl-tRNA synthetase and sterol 14α-demethylase. Table 5 lists the analysis results. A low binding energy between compounds and proteins typically indicates a high binding affinity between compounds and proteins or high inhibitory effects of the compounds on enzymes [31]. In this study, luteolin exhibited the highest binding energy (−106.9 kcal/mol) against tyrosinase, followed by gallic acid and quercetin. Manzoor et al. (2019) revealed that luteolin exhibited strong anti-tyrosinase activity; it inhibited the expression of cyclic adenosine monophosphate and the activity of adenyl cyclase through the α-MSH pathway [32]. In the present study, ellagic acid exhibited the highest binding energies against elastase, hyaluronidase, and COX-1 (−96.5, −102.5, and −104.8 kcal/mol, respectively). Moon et al. (2018) reported that ellagic acid exhibited strong antiaging activity; it activated both TGF-β1 and Wnt signaling pathways [33]. Baradaran Rahimi et al. (2020) also revealed that ellagic acid was the major active compound in pomegranate with antiaging and anti-inflammatory activities [34]. In the present study, quercetin exhibited the highest binding energies against collagenase, MMP-1, and tyrosyl-tRNA synthetase (−98.8, −109.2, and −118.2 kcal/mol, respectively). Quercetin is regarded as an excellent antiaging active ingredient in plant extracts [35], and it possesses broad-spectrum antibacterial properties (an antibacterial agent inhibiting tyrosyl-tRNA synthetase) [36]. In the present study, kaempferol exhibited the highest binding energies against COX-2 and TNF-α (−109.4 and −113.8 kcal/mol, respectively). Its anti-inflammatory properties are well documented [37]. Moreover, 3-*O*-methylellagic acid exhibited the highest binding energy (−105.3 kcal/mol) against sterol 14α-demethylase, thus demonstrating high antifungal activity [38].

**Figure 6:**
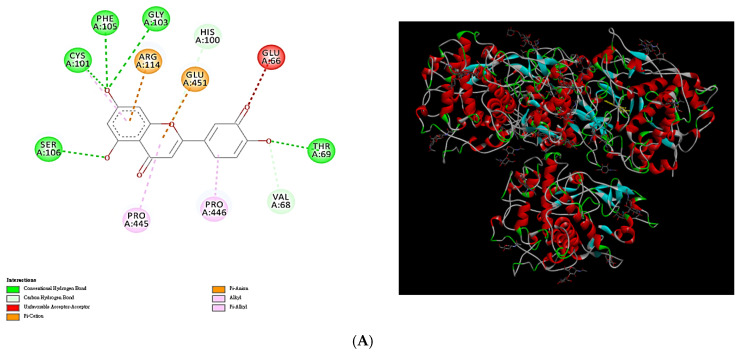

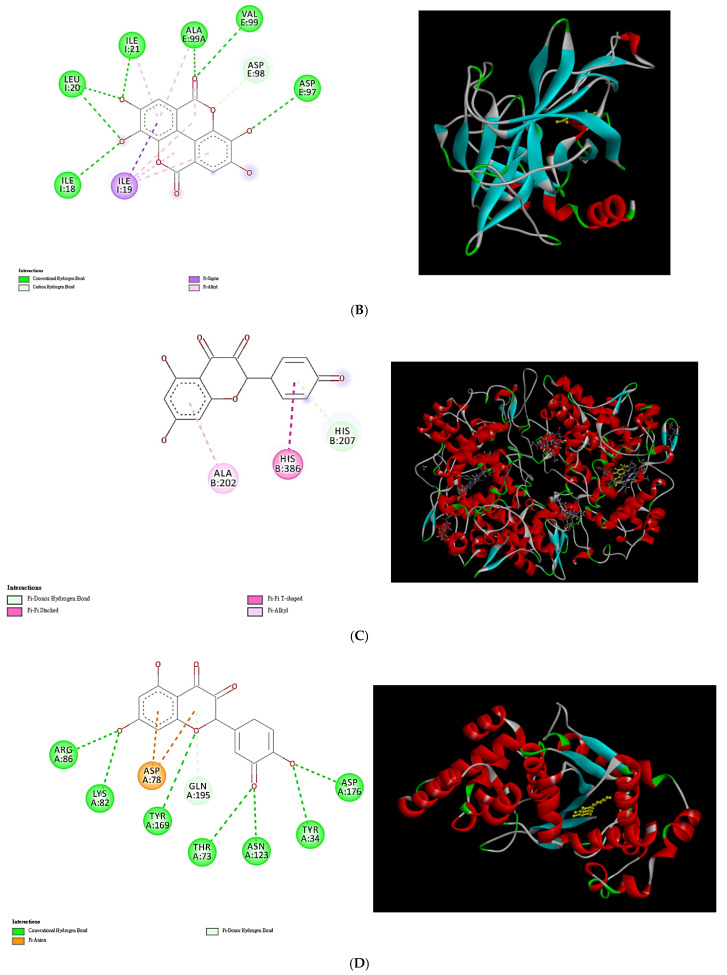

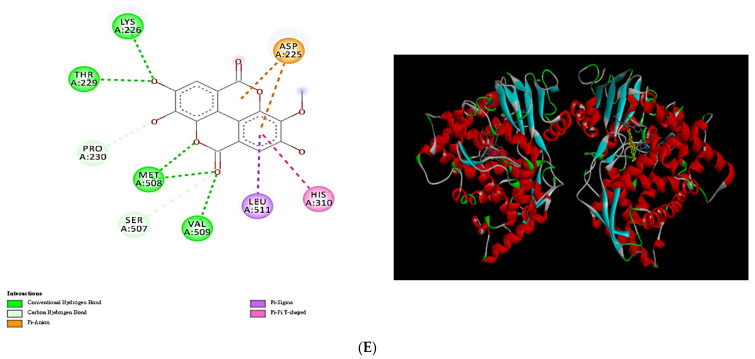
Docking interactions and docking complex of the optimal phytochemical compounds in the MLE (obtained through MAWE) against tested enzymes or proteins: (**A**) molecular docking of the interactions between luteolin and tyrosinase; (**B**) molecular docking of the interactions between ellagic acid and elastase; (**C**) molecular docking of the interactions between kaempferol and COX-2; (**D**) molecular docking of the interactions between quercetin and tyrosyl-tRNA synthetase; (**E**) molecular docking of the interactions between 3-*O*-methylellagic acid and sterol 14α-demethylase.

Figure 6 illustrates the docking interactions and docking complexes formed by selected phytochemical compounds in the MLE against tested enzymes. This study investigated the docking interactions of luteolin, ellagic acid, kaempferol, quercetin, and 3-*O*-methylellagic acid against tyrosinase, elastase, COX-2, tyrosyl-tRNA synthetase, and sterol 14α-demethylase, respectively. Among the compounds in the MLE, luteolin exhibited antityrosinase properties owing to the formation of H bonds with Ser106, Cys101, Phe105, Gly103, His100, Thr69, and Val68; π-cation bonds with Arg114; unfavorable acceptor–acceptor interactions with Glu66; π-anion bonds with Glu451; alkyl bonds with Pro446; and π-alkyl bonds with Pro445 and Cys101 (Figure 6A). Luteolin formed various molecular bonds with tyrosinase, facilitating the formation of protein–ligand complexes. Ellagic acid exhibited anti-elastase properties owing to the formation of H bonds with I1e8, Leu20, Ile22, Ala99, Val99, Asp98, and Asp97; π-σ bonds with Ile19; and π-alkyl bonds with Ile21, Ala99, and Ile19 (Figure 6B). Kaempferol exhibited anti-COX-2 properties owing to the formation of H bonds with His207, π-π bonds with His386, and π-alkyl bonds with Ala202 (Figure 6C). Quercetin exhibited anti-tyrosyl-tRNA synthetase activity mainly owing to the formation of H bonds with Arg86, Lys82, Tyr169, Thr73, Asn123, Tyr34, Asp176, and Gln195 and the formation of π-anion bonds with Asp78 (Figure 6D). In addition, 3-*O*-methylellagic acid exhibited anti-sterol 14α-demethylase activity owing to the formation of H bonds with Lys226, Thr229, Met508, Val509, Pro230, and Ser507; π-anion bonds with Asp225; π-σ bonds with Leu511; and π-π bonds with His310 (Figure 6E). On the basis of these results, we confirmed the relationship between the various physiological activities of the MLE and its active constituents. Nonetheless, it is important to note that these findings will require validation through future experiments utilizing pure chemical substances to inhibit the enzymes.

## 3. Materials and Methods

### 3.1. Plant Material and Extraction Procedure

*M. quinquenervia* leaves were collected from Nangang District, Taipei City, Taiwan (25°03′22″ N, 121°60′96″ E); the collected leaves were identified by Professor Bau-Yuan Hu. Voucher specimens (accession no. 20230215) were deposited in the herbarium of China University of Science and Technology, Taiwan. The collected leaves were washed with distilled water and dried in an oven (Eyela, Tokyo, Japan) at 50 °C for 2 h. The dried leaves were pulverized to a powder, which was passed through a 0.5 mm mesh. A sample of the powder (10 g) was extracted with various volumes of distilled water at LSRs of 10–30 mL/g. This extraction process was conducted in a microwave digestion instrument (SINEO, Shanghai Sineo Microwave Chemistry Technology Co., Ltd., Shanghai, China) operated at a power/frequency of 700 W/2.45 GHz; the extraction temperature was set to 80 °C and the irradiation time was 120 s. The optimal irradiation time was determined by extracting the sample with distilled water at an LSR of 20 mL/g in the microwave digestion instrument operated at an extraction temperature of 80 °C for various irradiation periods (60–300 s). The crude extracts were filtered through a Whatman filter (0.45 μm) and lyophilized using a shelf freeze-dryer (Uniss Corp., Taipei City, Taiwan) for the subsequent analysis of physiological activities.

### 3.2. Microbial Strains, Cells, and Reagents

Four bacterial strains, namely, ATCC 6538 (*Staphylococcus aureus*), ATCC 8739 (*Escherichia coli*), ATCC 9027 (*Pseudomonas aeruginosa*), and ATCC 6919 (*Cutibacterium acnes*), and two fungal strains, namely, ATCC 10231 (*Candida albicans*) and ATCC 16404 (*Aspergillus brasiliensis*), were employed for the current study; they were purchased from the Bioresource Collection and Research Center (BCRC; Hsinchu, Taiwan). The *S. aureus*, *E. coli*, and *P. aeruginosa* strains were cultured in tryptic soy broth (TSB) (DIFCO, Tucker, GA, USA) under aerobic conditions. The *C. acnes* strain was cultured in brain heart infusion broth (BHI) (DIFCO, Tucker, GA, USA) under anaerobic conditions. The *C. albicans* and *A. brasiliensis* strains were cultured in potato dextrose broth (PDB) (DIFCO, Tucker, GA, USA). The human skin fibroblast cell line CCD966SK (BCRC 60153) and the murine macrophage cell line RAW264.7 (BCRC 60001) were obtained from the BCRC. Normal human primary epidermal melanocytes neonatal (HEMn; C-102-5C) were obtained from Cascade Biologics (Portland, OR, USA). CCD966SK and HEMn cells were cultured in minimum essential medium containing 10% fetal bovine serum (FBS) and in Medium 254 with human melanocyte growth supplement (HMGS) (Thermo, Waltham, MA, USA), respectively. RAW264.7 cells were cultured in RPMI 1640 supplemented with 10% FBS and 1% penicillin–streptomycin (Thermo, Waltham, MA, USA). The chemicals used in this study were of analytical grade (purity ≥99.2%) and were purchased from Sigma-Aldrich (St. Louis, MO, USA).

### 3.3. Evaluation of Antioxidant Activity

The TPC of the derived MLE was determined using the Folin–Ciocalteu method, and TPC is expressed as gallic acid equivalents, as estimated using the method of Kujala et al. (2000) [39]. The TFC of the extracts was determined using the aluminum chloride colorimetric method [40] and is expressed as milligrams of rutin equivalents per gram of dry weight.

The antioxidant activity of the MLE was assessed in accordance with the protocols of Wu et al. (2018), Merchán-Arenas et al. (2011), and Lee et al. (2012) by using the DPPH free radical, ABTS free radical, and BCB assays, respectively [41,42,43]. For the DPPH, ABTS, and BCB assays, absorbance was recorded at 517, 734, and 470 nm on an ultraviolet–visible spectrophotometer (UV-2600i, Shimadzu, Kyoto, Japan), respectively. Ascorbic acid was used as a positive control for the DPPH assay. BHT was used as a positive control for the ABTS and BCB assays.

### 3.4. Extracellular and Intracellular Antityrosinase Activity

The extracellular antityrosinase activity of the MLE was assessed using the method of Zheng et al. (2012) [44]. In brief, the freeze-dried leaf extract was dissolved in dimethyl sulfoxide (DMSO) to obtain an MLE with concentrations of 0–200 mg/L. Moreover, 30 μL of the sample was mixed with 970 μL of 0.05 mM phosphate-buffered saline (PBS). Subsequently, 1 mL of 100 mg/L L-tyrosine and 1 mL of 350 U/mL mushroom tyrosinase solution were added to the sample and reacted in the dark for 20 min. After completion of the reaction, the absorbance of the solution was measured at 490 nm. Positive controls were α-arbutin and kojic acid, a commercial whitening agent. The antityrosinase activity of the MLE was calculated using the following formula:

(1) $$\mathrm{Antityrosinase}\;\mathrm{activity}\;(\%)=\frac{\left[(A-B)-(C-D)\right]}{\left(A-B\right)}\times 100$$

where A is the OD_490_ of the control (without the MLE), B is the OD_490_ of the blank of A (without the MLE or tyrosinase), C is the OD_490_ of the experimental group (with the MLE and tyrosinase), and D is the OD_490_ of the blank of C (without tyrosinase).

The intracellular antityrosinase activity of the MLE and the melanin content of the HEMn cells were analyzed using the method of Wu et al. (2018) [41]. In brief, the HEMn cells were seeded in 24-well plates at a density of 3 × 10^5^ cells/well and cultured with 2.5 mL Medium 254 supplemented with 1% HMGS at 37 °C under 5% CO_2_. After 24 h of cultivation, the cells were treated with the MLE (0–200 mg/L) for another 24 h. Subsequently, the cells were washed with PBS, lysed with lysis buffer, and sonicated using an ultrasonic sonicator (Qsonica, Newtown, CT, USA). To determine the intracellular antityrosinase activity of the MLE, the lysate was collected and reacted with 1.25 mM l-dopa for 3 h, and the absorbance of the solution was measured at 475 nm on an Epoch ELISA reader (BioTek Instruments, Santa Clara, CA, USA). To measure the melanin content of the HEMn cells, the sonicated product was further centrifuged at 8000× *g* for 10 min by using a micro-ultracentrifuge (Thermo Fisher Scientific, Waltham, MA, USA). The pellets were then dissolved in 1 N NaOH solution containing 10% DMSO and reacted for 30 min at 80 °C. Absorbance was measured at 405 nm on the ELISA reader. We quantified the melanin content of the HEMn cells by using a calibration curve plotted with OD_405_ values of synthetic melanin versus varying concentrations of synthetic melanin.

### 3.5. Cytotoxicity Assay

The viability of the HEMn, CCD966SK, and RAW264.7 cells was determined using the 3-(4,5-dimethylthiazol-2-yl)-2,5-diphenyltetrazolium bromide (MTT) colorimetric assay [45]. These cells were seeded in 96-well microplates at a density of 5 × 10^5^ cells/well and cultured at 37 °C under 5% CO_2_. The next day, the culture medium was removed, and 0–500 mg/L MLE in fresh medium was added to the wells. After 24 h of incubation, MTT solution (5 mg/mL in PBS) was added to the cells and reacted for 2 h. Subsequently, 0.1 mL of DMSO was added to each well to stop the reaction and solubilize the resulting formazan crystals. The absorbance of the final solution was measured at 570 nm on the ELISA reader. Cell viability (%) was estimated as the percentage of the absorbance of the sample with the MLE relative to the absorbance of the blank (without the MLE).

### 3.6. Evaluation of Antiaging Activity

The antiaging activity of the MLE was evaluated by assessing its inhibitory effects on MMP-1, collagenase, elastase, and hyaluronidase activities. The activity of MMP-1 in the CCD966SK cells was assessed using the human MMP-1 ELISA kit (RayBiotech, Norcross, GA, USA), as described by Chen et al. (2022) [46]. In brief, CCD-966SK cells were cultured in 96-well plates for 24 h in a 5% CO_2_ atmosphere at 37 °C. Subsequently, the culture media were discarded, and varying concentrations of MLE were introduced to each well for an additional 24 h. The ELISA kit facilitated the mixing of components, and the reaction was conducted at 25 °C. After a 2 h incubation period, the resultant mixture was analyzed spectrophotometrically at 420 nm. Additionally, extracellular collagenase activity was assessed using the modified fluorogenic DQ gelatin degradation assay, in accordance with the method of Li et al. (2020) [47]. Briefly, varying concentrations of MLE were added to a 96-well plate, along with 1 U/mL collagenase (100 μL per well) and 15 μg/mL DQ gelatin. The mixture was allowed to react for 20 min, after which the absorbance was recorded at 485 nm and 530 nm (excitation and emission wavelengths, respectively) to assess the rate of gelatin proteolysis. Extracellular elastase activity was evaluated using porcine pancreatic elastase as a model enzyme, with N-succinyl-Ala-Ala-Pro-Val-*p*-nitroanilide (Suc-Ala) serving as the substrate [41]. In this procedure, 50 μL of MLE at varying concentrations was combined with 125 μL of 7 mM Suc-Ala (pH 8.0, prepared in 0.1 M Tris–Cl buffer) and incubated for 15 min at 25 °C in a 96-well plate. Following this, 25 μL of 0.3 U/mL neutrophil elastase was added, and the reaction continued for an additional 15 min. The absorbance was subsequently measured at 405 nm using the Epoch ELISA reader. Finally, extracellular hyaluronidase activity was evaluated using the Hyaluronidase Enzymatic Assay Kit (Sigma-Aldrich, St. Louis, MO, USA), employing a spectrophotometric method with hyaluronidase as the enzyme and hyaluronic acid as the substrate [48]. The hyaluronidase activity was quantified by measuring the absorbance at 600 nm with the Epoch ELISA reader. EGCG, gallic acid, or oleanolic acid was used as a positive control in the antiaging assay if required.

### 3.7. Evaluation of Anti-Inflammatory Activity

The anti-inflammatory activity of the MLE was evaluated through both enzyme-based and cell-based assays. The inhibitory effects of the MLE on the activities of the proinflammatory enzymes COX-1 and COX-2 were assessed using a colorimetric COX inhibitor screening assay kit (Cayman, Ann Arbor, MI, USA) according to the manufacturer’s instructions. In the cell-based assay, the anti-inflammatory activity of the MLE in RAW264.7 cells was examined by inducing inflammatory responses in the cells by using a lipopolysaccharide. Moreover, TNF-α was used as an indicator of anti-inflammatory activity, and its levels were determined using commercial ELISA kits (R&D systems Inc., Minneapolis, MN, USA) according to the manufacturer’s instructions. Indomethacin was used as a positive control in the assays.

### 3.8. Evaluation of Antimicrobial Activity

The MIC and MFC of the MLE were employed as indicators of its antibacterial and antifungal activities, respectively. The antibacterial activity of the MLE in the *E. coli*, *S. aureus*, *P. aeruginosa*, and *C. acnes* strains was evaluated using the tube dilution method [49]. In brief, a test tube was prepared by combining 1 mL of MLE, with its concentration being adjusted as necessary; 2 mL of TSB for *E. coli*, *S. aureus*, and *P. aeruginosa* or BHI for *C. acnes*; and 1 mL of inoculum at a concentration of 2 × 10^7^ cfu/mL. The incubation conditions varied according to the bacterial species; the test tubes containing *E. coli*, *S. aureus*, or *P. aeruginosa* were incubated for 18 h at 37 °C under aerobic conditions, while the test tube containing *C. acnes* was incubated for 48 h at 37 °C under anaerobic conditions. MIC refers to the lowest concentration of a chemical/compound that prevents cell growth. The antifungal activity of the MLE in the *C. albicans* and *A. brasiliensis* strains was determined using the conventional plate count method [46]. In brief, a mixture was prepared by combining 1 mL of MLE, with its concentration being adjusted, 1 mL of inoculum at a concentration of 8 × 10^6^ cfu/mL, and 100 mL of PDB in a conical flask. This mixture was subsequently incubated for 5 days at 25 °C. MFC refers to the lowest extract concentration of a chemical/compound at which no visible growth of the subculture occurs. Streptomycin, a broad-spectrum bactericidal agent, was used as a positive control against *E. coli*, *S. aureus*, and *P. aeruginosa*. Erythromycin, which is frequently used for acne vulgaris treatment, was used as a positive control against *C. acnes*. Nystatin, an antifungal agent, was used as a positive control against *C. albicans* and *A. brasiliensis*.

### 3.9. Quantification of Chemical Compounds in MLE

The primary chemical compounds in the MLE were analyzed through GC-MS (Shimadzu, Kyoto, Japan), in accordance with the method of Chao et al. (2017) [1]. In brief, GC-MS was applied in the EI mode at 70 eV with a mass range of *m*/*z* 35–500. A DB-5 fused capillary column (30 m × 0.25 mm i.d.) with a thickness of 0.25 μm for the coated material was used. The injector and detector temperatures were set at 280 and 300 °C, respectively. The temperature program was as follows: the temperature was initially maintained at 35 °C for 3 min, increased at a rate of 5 °C/min to 300 °C, and then maintained at 300 °C for 10 min. The flow rate of the carrier gas (helium) was maintained at 1.5 mL/min. The chromatographic retention index (RI) was calculated by referring to a homologous series of *n*-alkanes (C_6_–C_22_), and GC-MS was conducted in accordance with the previously specified conditions. The chemical compounds were quantified using the percentage relative peak area and identified by referring to the chromatographic peaks in a standard library from the National Institute of Standards and Technology (NIST 20) MS spectral database. The chemical compounds in the MLE were confirmed by comparing their RI values with those of authentic compounds.

The main phenolic acids and flavonoids in the MLE were analyzed through HPLC (Hitachi, Tokyo, Japan), in accordance with the method of Trabelsi et al. (2013) [50], with slight modifications. In brief, a prontosil C18 column (250 mm × 4.0 mm × 5 μm) was used for HPLC. The mobile phase was composed of two solvents: 0.025% trifluoroacetic acid in H_2_O (A) and acetonitrile (B). The elution program at a flow rate of 1 mL/min was as follows: 15% B, followed by 20% B at 5 min, 26% B at 10 min, 38% B at 15 min, 50% B at 20 min, 100% B at 25 min, and 15% B at 30 min. The injection volume was 20 μL and peaks in the chromatogram were monitored at 280 nm. The peaks were identified through comparison with those of standard samples under the same conditions. To compare the total concentrations of phenolic acids and flavonoids identified by HPLC with TPC and TFC in the MLE, the concentrations of individual phenolic acids and flavonoids were initially quantified using standard curves that correlate the concentrations of standard samples with their corresponding peak area values. Following this, the concentrations of each phenolic acid and flavonoid were assessed utilizing the Folin–Ciocalteu method [39] and the aluminum chloride colorimetric method [40].

### 3.10. Molecular Docking Study

To examine the possible mechanisms underlying the various physiological activities of the MLE, the 10 main phytocompounds found in the MLE were subjected to a molecular docking study. The molecular docking study was conducted using iGEMDOCK V2.1 software with the following parameters: population size = 200, generations = 70, and number of solutions = 2. The 3D chemical structures of selected compounds were docked against the active sites of tyrosinase, elastase, collagenase, hyaluronidase, MMP-1, COX-1, COX-2, TNF-α, tyrosyl-tRNA synthetase, and sterol 14α-demethylase (CYP51). The 3D chemical structures of selected compounds and the crystal structures of target proteins were obtained from the PubChem database and the Protein Data Bank database, respectively. The best match was chosen on the basis of the total binding energy, which is the sum of the energy of the hydrogen bond, van der Waals forces, and electrostatic interactions.

### 3.11. Statistical Analysis

The study data were assessed using a one-way analysis of variance, followed by Duncan’s multiple range test. The data are expressed as means ± standard deviations (*n* = 3). A *p* value of <0.05 was considered indicative of statistical significance. The IC_50_ was calculated using GraphPad Prism software (version 9) (GraphPad Software, San Diego, CA, USA). All statistical analyses were performed using IBM SPSS (version 26) (IBM Corp., Armonk, NY, USA).

## 4. Conclusions

This study applied MAWE to produce an MLE and comprehensively demonstrated its physiological or pharmacological activities. The MLE obtained under optimal extraction conditions exhibited a higher yield and superior physiological activities compared with extracts obtained through conventional extraction techniques. Moreover, our study is the first to reveal the antiaging and anti-inflammatory activities of the MAWE-derived MLE. The MAWE technique is environmentally friendly because it uses water as the extraction solvent. Furthermore, the MLE was noted to exhibit low cytotoxicity and broad-spectrum physiological activities, indicating that it has potential for use in cosmetics, food, medicine, and other products. A molecular docking analysis of the primary constituents of the MLE revealed that luteolin exhibited optimal skin-whitening effects, ellagic acid exhibited excellent antiaging and anti-inflammatory activities, quercetin exhibited favorable antiaging and antibacterial activities, kaempferol exhibited excellent anti-inflammatory activity, and 3-*O*-methylellagic acid exhibited the highest antifungal activity. Thus, these potential active ingredients can be further extracted and purified for use in various industries.

## Figures and Tables

**Table 1:** Antiaging activity (IC_50_ values) of the MLE obtained through MAWE under optimal conditions *.

Tested Sample	MMP-1 Activity	Collagenase Activity	Elastase Activity	Hyaluronidase Activity
*M. quinquenervia* leaf extract	114.8 ± 8.1	187.2 ± 5.4	73.4 ± 2.4	60.4 ± 3.1
EGCG	42.3 ± 3.1	113.7 ± 9.1	93.5 ± 7.2	382 ± 12.6
Gallic acid	–	126.8 ± 3.7	–	–
Oleanolic acid	–	–	78.2 ± 1.8	98.6 ± 5.2

* LSR, 20 mL/g; extraction temperature, 80 °C; microwave irradiation time, 180 s; and microwave irradiation power, 700 W.

**Table 2:** Antimicrobial activity of the MLE obtained through MAWE under optimal conditions *.

Tested Sample	MIC				MFC	
	*S. aureus*	*E. coli*	*P. aeruginosa*	*C. acnes*	*C. albicans*	*A. brasiliensis*
*M. quinquenervia* leaf extract	64	64	128	128	256	128
Streptomycin	64	32	32	–	–	–
Erythromycin	–	–	–	8	–	–
Nystatin	–	–	–	–	32	16

* LSR, 20 mL/g; extraction temperature, 80 °C; microwave irradiation time, 180 s; and microwave irradiation power, 700 W.

**Table 3:** Composition of the main chemicals and their relative content in the volatile part of the MLE obtained through MAWE under optimal conditions *.

No.	RI	Chemical Compounds	Categories	Relative Content (%)
1	934	α-pinene	monoterpene	10.61
2	952	camphene	monoterpene	0.62
3	961	benzaldehyde	hydrocarbons	0.74
4	978	β-pinene	monoterpene	7.52
5	990	myrcene	monoterpene	0.52
6	1025	*p*-cymene	aromatic compounds	2.45
7	1029	o-cymene	monoterpene	0.75
8	1034	limonene	monoterpene	7.94
9	1042	1,8-cineole	monoterpene	16.71
10	1062	γ-terpinene	monoterpene	1.52
11	1092	terpinolene	monoterpene	2.14
12	1102	linalool	monoterpene	0.67
13	1178	4-terpineol	monoterpene	1.55
14	1205	α-terpineol	monoterpene	10.12
15	1352	α-terpinyl acetate	monoterpene	2.51
16	1428	caryophyllene	sesquiterpene	2.17
17	1465	α-humulene	sesquiterpene	1.05
18	1469	β-humulene	sesquiterpene	0.61
19	1501	ledene	sesquiterpene	8.52
20	1507	α-selinene	sesquiterpene	0.74
21	1567	nerolidol	sesquiterpene	1.81
22	1596	viridiflorol	sesquiterpene	13.27
23	1610	ledol	sesquiterpene	1.23
24	1615	globulol	sesquiterpene	2.67

* LSR, 20 mL/g; extraction temperature, 80 °C; microwave irradiation time, 180 s; and microwave irradiation power, 700 W.

**Table 4:** Composition of the main phenolic compounds and their content in the MLE obtained through MAWE under optimal conditions *.

Chemical Compounds	Contents
Phenolic acids (mg GAE/g DW)	
gallic acid	104.2
vanillic acid	10.2
caffeic acid	28.6
ferulic acid	8.1
rosmarinic acid	6.3
ellagic acid	88.1
3-O-methyl ellagic acid	58.6
Flavonoids (mg RE/g DW)	
rutin	1.7
luteolin	23.1
catechin	3.8
quercetin-3-*O*-glucuronopyranoside	2.8
kaempferol-3-*O*-glucoside	3.2
quercetin	13.8
apigenin	2.4
naringin	1.2
kaempferol	16.3
hesperidin	1.8

* LSR, 20 mL/g; extraction temperature, 80 °C; microwave irradiation time, 180 s; and microwave irradiation power, 700 W.

**Table 5:** Results of the molecular docking analysis of the 10 main phytocompounds in the MLE obtained through MAWE under optimal conditions *.

	Gallic Acid	Ellagic Acid	3-*O*- Methylellagic Acid	Luteolin	Quercetin	Kaempferol	α-Pinene	1,8-Cineole	α-Terpineol	Viridiflorol
	Total binding energy (kcal/mol)									
tyrosinase	−101.6	−92.3	−94.2	−106.9	−98.2	−96.2	−49.7	−52.3	−62.9	−83.2
elastase	−80.7	−96.5	−83.3	−88.9	−87.4	−91.8	−55.5	−62.3	−61.3	−64.7
collagenase	−78.4	−91.8	−92.6	−94.2	−98.8	−95.1	−56.6	−60.7	−69.8	−73.6
hyaluronidase	−78.0	−102.5	−96.2	−90.1	−99.9	−95.6	−55.2	−57.9	−59.2	−64.6
MMP-1	−93.3	−95.6	−87.9	−98.6	−109.2	−103.6	−56.3	−55.7	−70.1	−72.6
COX-1	−85.8	−104.8	−100.2	−92.3	−96.8	−93.8	−57.6	−60.5	−66.5	−77.5
COX-2	−81.7	−103.4	−104.1	−102.1	−101.7	−109.4	−55.6	−57.4	−70.4	−72.8
TNF-α	−80.3	−100.7	−102.6	−90.6	−90.4	−113.8	−51.2	−60.9	−65.6	−77.3
tyrosyl-tRNA synthetase	−82.0	−110.3	−108.0	−95.5	−118.2	−88.9	−49.2	−54.8	−57.5	−66.7
sterol 14α-demethylase	−88.1	−95.8	−105.3	−98.4	−98.1	−90.2	−50.3	−54.1	−62.3	−67.8

* LSR, 20 mL/g; extraction temperature, 80 °C; microwave irradiation time, 180 s; and microwave irradiation power, 700 W.

## Data Availability

All data generated or analyzed during this study are included in this published article.
